# Synthesis, enzyme inhibition, and docking studies of new schiff bases of disalicylic acid methylene-based derivatives as dual-target antibacterial agents

**DOI:** 10.3389/fchem.2024.1493906

**Published:** 2024-11-12

**Authors:** Lamya H. Al-Wahaibi, Mohamed A. Mahmoud, Hayat Ali Alzahrani, Hesham A. Abou-Zied, Alshaimaa Abdelmoez, Bahaa G. M. Youssif, Stefan Bräse, Safwat M. Rabea

**Affiliations:** ^1^ Department of Chemistry, College of Sciences, Princess Nourah bint Abdulrahman University, Riyadh, Saudi Arabia; ^2^ Pharmaceutical Organic Chemistry Department, Faculty of Pharmacy, Assiut University, Assiut, Egypt; ^3^ Applied Medical Science College, Medical Laboratory Technology Department, Northern Border University, Arar, Saudi Arabia; ^4^ Medicinal Chemistry Department, Faculty of Pharmacy, Deraya University, Minia, Egypt; ^5^ Institute of Biological and Chemical Systems - Functional Molecular Systems (IBCS-FMS), Karlsruhe Institute of Technology, Karlsruhe, Germany; ^6^ Medicinal Chemistry Department, Faculty of Pharmacy, Minia University, Minia, Egypt; ^7^ Apogee Pharmaceuticals, Burnaby, BC, Canada

**Keywords:** bacterial resistance, biofilm, DNA, isatin, salicylic acid, bactericidal

## Abstract

**Introduction:**

Bacteria have acquired resistance to almost all antibiotics currently in use due to their extensive, broad, and improper utilization over a prolonged period. DNA gyrase and DHFR exhibit significant promise as targets for antibacterial therapeutics.

**Methods:**

We have developed a series of disalicylic acid methylene/Schiff bases hybrids (**6a-l**) that function as antibacterial agents by targeting DNA gyrase and DHFR.

**Results and discussion:**

The findings showed that **6a-l** have significant antibacterial activity against both Gram-positive and Gram-negative bacteria, with inhibition zones (IZ) comparable to or even higher than the reference Ciprofloxacin. MIC testing revealed that **6h** and **6l** were 1.5 times as effective than ciprofloxacin against *S. aureus*. Compounds **6h** and **6l** had MBC values of 28 and 33 nM for *S. aureus*, compared to Ciprofloxacin’s 45 nM, indicating that they are more potent bactericidal agents. The MIC values for compounds **6c**, **6e**, **6h**, **6j**, and **6l** against *A. flavus* were between 14.50 and 19.50 µM, while the MIC value for fluconazole was 11.50 µM. Also, the studied compounds had MIC values between 18.20 and 22.90 µM against *C. albicans*, while Fluconazole had a MIC value of 17.50 µM. Compound **6h** showed a MIC value of 1.70 µM against the clinical strain *S. aureus* (ATCC 43300) (MRSA), making it an effective antibacterial agent. Compounds **6h**, **6j**, and **6l** inhibited *E. coli* DNA gyrase with IC_50_ values of 79, 117, and 87 nM, respectively, compared to the reference novobiocin (IC_50_ = 170 nM). Additionally, compounds **6h** and **6l**, the most potent *E. coli* gyrase inhibitors, showed encouraging results on DHFR. Compounds **6h** and **6l** exhibit IC_50_ values of 3.80 µM and 4.25 µM, respectively. These values are significantly lower and hence more effective than Trimethoprim’s IC_50_ of 5.20 µM.

## 1 Introduction

Bacterial infections, caused by either Gram-positive or Gram-negative pathogens, are the most common type of infections acquired in hospitals or by the general public (Chu et al., 2019; Abdel-Aziz et al., 2023). Moreover, bacteria have developed resistance to nearly all currently used antibiotics as a result of their long-term, widespread, and incorrect use, complicating the situation (Gao C. et al., 2018; Gao F. et al., 2018; Kumar et al., 2023a). Annually, some 0.7 million deaths occur worldwide due to drug-resistant infections, and this figure might rise to 10 million by the year 2050 if current patterns persist (Agrawal and Patel, 2024; Kumar et al., 2023b). Therefore, it is imperative to expedite the development of novel antibacterials that demonstrate exceptional efficacy against both susceptible and resistant infections (Kumar et al., 2022).

The enzyme dihydrofolate reductase (DHFR) is a crucial target for numerous anticancer and antibacterial medications. It holds significant value in the field of medicinal chemistry due to its role as a cofactor in the production of nucleic acids and amino acids (Roth, 1986; Abdolmaleki and Maddah, 2023). The mechanism of action for DHFR involves the inhibition of DNA, RNA, and protein synthesis, which leads to the stop of cell growth (Alrohily et al., 2019; Chawla et al., 2021). On the other hand, DNA gyrase is a type II topoisomerase enzyme that catalyzes modifications in the topology of DNA (Abdel-Aziz et al., 2023; Hirsch and Klostermeier, 2021). Furthermore, it consists of two chains, GyrA and GyrB subunits, which are accountable for the temporary disruption of two DNA strands and the induction of negative supercoiling in DNA during replication. Antibacterial drugs that specifically target DNA gyrase exert their activity through two mechanisms: gyrase poisoning, as seen in ciprofloxacin, or by blocking the ATP binding site, as observed in novobiocin (Tiz et al., 2019). Due to its crucial role, DNA gyrase has become an appealing target for the development of antibacterial drugs (Elbastawesy et al., 2023). As a result, DHFR and DNA topoisomerases have a demonstrated track record of supporting their function in microbial disorders (Hassan et al., 2020; Alqurashi et al., 2023).

The development of biofilms is another contributing component to bacterial resistance’s pathogenicity. Biofilms consist of an intricate community of bacteria enclosed within a polysaccharide matrix (Singh et al., 2021; Shree et al., 2023). A significant portion of bacterial pathogens generate biofilms as a pathogenic mechanism to adhere to surfaces and protect themselves from antimicrobial agents. More than 80% of persistent microbial infections are associated with biofilms, which pose a significant health concern (Vestby et al., 2020). As a result, given the significant role biofilms play in infection transmission and resistance evolution, there is an urgent need for the discovery of new chemotherapeutic drugs that interfere with biofilm formation and/or break pre-formed ones.

**FIGURE 1:**
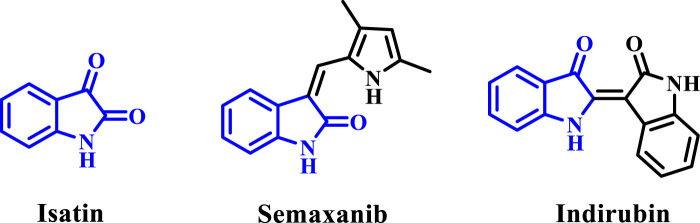
Structures of isatin, semaxanib, and indirubin.

The investigation of medically advantageous heterocyclic frameworks is an essential arena in the field of drug discovery. The compound is referred to as isatin (1*H*-indole-2,3-dione, Figure 1) moiety is widely present in nature, and its derivatives exhibit a wide range of pharmacological effects, with anti-bacterial activity (Zhang et al., 2018; Liu et al., 2022) being the most prominent.

The isatin moiety offers a wide range of modification possibilities, with the N-1, C-3, and C-5 positions being the most important ones for chemical modification (Wang et al., 2018). Additionally, a number of isatin-based medications, such as Semaxanib and Indirubin, are currently in clinics or undergoing clinical trials for the treatment of a variety of disorders (Xu et al., 2017a; Xu et al., 2017b). The extensive variety of biological activities, along with the ability to make various structural alterations, and the effective use in clinical practice, have motivated researchers to investigate isatins and develop many derivatives with different structures.

**FIGURE 2:**
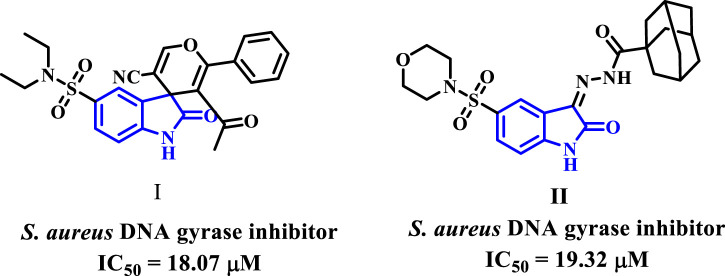
Isatin-based DNA gyrase inhibitors **I** and **II**.

Recently, researchers investigated various isatin compounds to establish their efficacy against bacteria. Some of these compounds (**I** and **II**, Figure 2) have demonstrated promise *in vitro* assays as DNA gyrase inhibitors (Alzahrani et al., 2022).

Schiff base structures are becoming increasingly popular among researchers due to their ease of synthesis, versatility, and diverse range of activities, including antibacterial properties (Bayeh et al., 2020). In addition, the imine bond found in Schiff base offers the potential to form interactions with various nucleophiles and electrophiles, thereby impeding the activity of enzymes or the replication of DNA (Nastasă et al., 2018).

The combination of multiple pharmacophores into a single hybrid molecule is a promising approach to the development of innovative drugs. This approach has the potential to overcome cross-resistance and increase the effectiveness of the original medications (Hisham et al., 2023; Frejat et al., 2022). In addition, many hybrids, such as **Ro 23-9424** and **TD-1792**, are currently undergoing clinical studies to help battle a variety of diseases. So, combining isatin with other antibacterial pharmacophores could result in very potent options that function against both drug-sensitive and drug-resistant Gram-positive and Gram-negative bacteria.

### 1.1 Rational design

**FIGURE 3:**
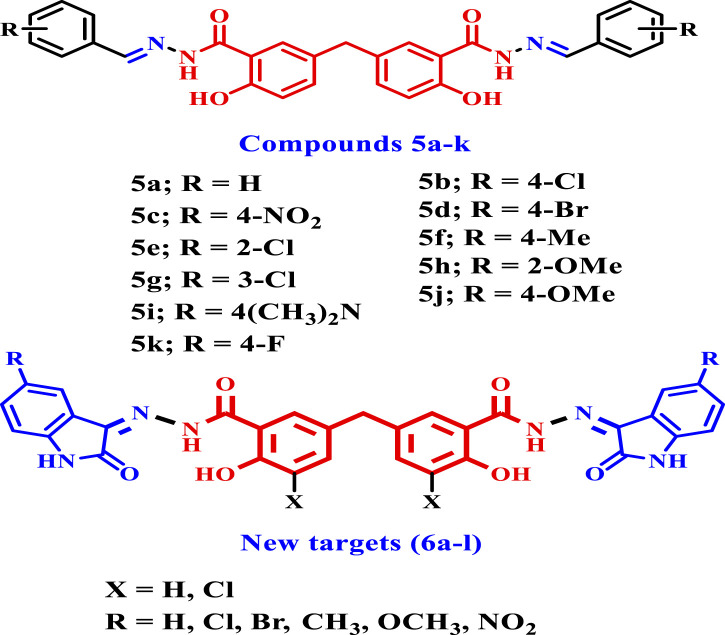
Structures of compounds **5a-k** and new targets **6a-l**.

In a recent work from our lab (Al-Wahaibi et al., 2024), we reported on the design, synthesis, and antibacterial activity of a new series of Schiff bases of disalicylic acid methylene hybrid with various aldehydes as DNA gyrase and Topoisomerase IV inhibitors. The novel hybrids **5a-k**, Figure 3, were tested for antibacterial efficacy against Gram-positive pathogens *S. aureus* (*S. aureus*) and *B. subtilis* (*B. subtilis*), as well as Gram-negative organisms *E. coli* (*E. coli*) and *P. aeruginosa* (*P. aeruginosa*). Ciprofloxacin was utilized as the reference medication.

The *in vitro* assay test showed that compound **5h** (X = 2-OMe) had the highest potency among the compounds examined, with MIC values of 0.030, 0.065, and 0.060 μg/mL against *S. aureus*, *E. coli*, and *P. aeruginosa*. It was as effective as ciprofloxacin against the studied species but had a MIC value of 0.050 μg/mL against *B. subtilis*, making it five times less potent than ciprofloxacin. Additionally, compound **5i** (X = 4-dimethylamino) exhibited the second greatest activity. At the MIC level, it was equally effective against *S. aureus*, *E. coli*, and *P. aeruginosa* as compound **5h** and ciprofloxacin were. However, against *B. subtilis*, it was 7 times less effective than ciprofloxacin. Additionally, the inhibitory efficacy of **5h** and **5i** against *E. coli* DNA gyrase was assessed using the *E. coli* DNA gyrase test. Compounds **5h** and **5i** exhibited greater inhibitory activity against *E. coli* DNA gyrase, with IC_50_ values of 92 ± 5 nM and 97 ± 6 nM, respectively, compared to the positive control novobiocin (IC_50_ = 170 nM). Compounds **5h** and **5i** also demonstrated promising effects on Topoisomerase IV. Compounds **5h** and **5i** exhibit IC_50_ values of 3.50 µM and 5.80 µM, respectively. These results are much lower and more potent than Novobiocin’s IC_50_ value of 11 µM.

In the present study, the newly synthesized compounds **(6a-l)** were developed by making two modifications to the previously disclosed **5a-k** compounds. Our first modification involves replacing aldehydes with isatin or isatin derivatives during the synthesis of our new hybrids. Second, in compounds **6j-l**, we use a dichloro-disalicylic acid methylene derivative rather than disalicylic acid methylene to increase the antibacterial activity.

Based on the data above and our ongoing research into developing medicinally active antimicrobials (Abdel-Aziz et al., 2023; Elbastawesy et al., 2023; Frejat et al., 2022; Al-Wahaibi et al., 2024; Al-Wahaibi et al., 2021a; Al-Wahaibi et al., 2021b; Aly et al., 2024; Abdu-Allah et al., 2017; Alkhaldi et al., 2018; Alkhaldi et al., 2019; Youssif, 2013; Hofny et al., 2021; Youssif et al., 2016), we report the design and synthesis of new isatin-based Schiff bases **6a–l** (Figure 3), obtained from methylene disalicylic acid hydrazide. The novel compounds **6a-l** were tested for antibacterial activity against Gram-positive pathogens *S. aureus* and *B. subtilis*, as well as Gram-negative organisms *E. coli* and *P. aeruginosa*. Ciprofloxacin was utilized as the reference medication. Furthermore, the synthetic compounds **6a-l** were tested for antifungal activity against Aspergillus flavus (*A. flavus*) and *Candida* albicans (*C. albicans*), using fluconazole as a reference medication. The minimum inhibitory concentrations (MICs), bactericidal concentrations (MBCs), and fungicidal concentrations (MFCs) of the most active derivatives against the tested microorganisms were compared to ciprofloxacin and/or fluconazole. This study looked at the antibacterial efficacy of the most potent Schiff bases against a panel of multi-drug resistant bacteria (MDRB), which included one clinical strain of *S. aureus* (ATCC 43300) (MRSA), two standard strains of *E. coli* (ATCC BAA-196), and *P. aeruginosa* (ATCC BAA-2111). Norfloxacin, a broad-spectrum antibiotic, was used as the positive control. Additionally, the evaluation focused on assessing the inhibitory potency of the most active compounds against *E. coli* DNA gyrase and DHFR, which were identified as potential targets. Finally, the cell viability and antibiofilm assays for the most potent compounds were evaluated.

## 2 Results and discussion

### 2.1 Chemistry

**SCHEME 1:**
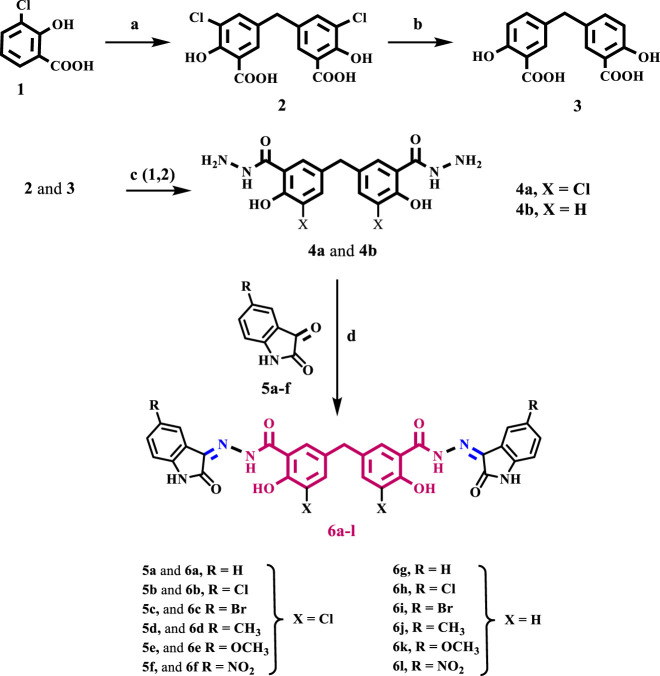
Reagents and reaction conditions: **(A)** HCHO, H2SO4, 35°C, 18 h, 98%; **(B)** KHCOO, Pd/C, KOH, 2- PrOH/H2O, 70°C, 4 h, 92%; **(C)** 1-EtOH/Conc. H2SO4, 2- Hydrazine hydrate, reflux 6 h; **(D)** isatin derivative, EtOH, reflux overnight.

Scheme 1 depicts the synthesis steps for the key intermediates **4a** and **4b**, as well as the target compounds **6a-l**. Compounds **2**, **3**, and **4b** were previously synthesized and well described (Al-Wahaibi et al., 2024). The procedures used in the current investigation differ slightly from those used in Cushman and Suseela’s study (Cushman and Kanamathareddy, 1990). The synthesis commenced by preparing the methylene bridged 3-chlorosalicylic acid dimer **2** (DSA-Cl_2_) with a yield of 98%. This was achieved through the condensation of two molecules of 3-chlorosalicylic acid **1** with paraformaldehyde in concentrated sulfuric acid. The chlorine atom was used as a protective group to control the regio-chemistry in the dimerization reaction and prevent further interaction of DSA-Cl_2_ with formaldehyde in the presence of acid. This interaction would lead to the formation of phenol-formaldehyde polymers.

The subsequent step involved the reduction of compound **2** through reductive dehalogenation using potassium formate as a source of hydride and Pd/C as a catalyst, resulting in the formation of disalicylic acid dimer **3** with a yield of 92%. Initially, the hydrodehalogenation of the chlorine atom in compound **2** to produce disalicylic acid dimer **3** was attempted using molecular hydrogen (Youssif, 2013). However, this reaction proceeded slowly and was incomplete even after 24 h. To overcome this, catalytic transfer hydrogenation (CTH) was employed, utilizing organic hydrogen donors such as potassium formate (HCOOK) as a reducing agent. CTH demonstrated a significantly higher reaction rate with potassium formate compared to molecular hydrogen. Additionally, potassium formate is easily manageable, less toxic, and less flammable, making it more advantageous for large-scale synthesis.

Compound **4a** was prepared from compound **2** using a two-step process. Firstly, compound **2** was heated in absolute ethanol with a catalytic amount of concentrated sulfuric acid. Then, the resulting ester product was further refluxed with an excess of hydrazine hydrate. Compound **4a** was obtained in a 65% yield as white crystals after purification. The structure of compound **4a** was confirmed using IR spectroscopy, which showed a strong absorption peak at υ 2,616–3,370 cm^−1^, of phenolic OH groups. In addition, the spectrum exhibited prominent absorption peaks at υ 3,370 and 3,286 cm^−1^, corresponding to the NH_2_ group. Additionally, the spectrum revealed a characteristic peak at υ 1,654 cm^−1^, corresponding to the amidic C=O bond. Unfortunately, due to solubility issues, we were unable to obtain a distinct ^1^H NMR or ^13^C NMR spectrum for **4a** using commonly used NMR solvents. We use LC-MS spectroscopy, which revealed the molecular ion peak of **4a** in the anticipated region of the mass spectrum (See Supplementary Material).

Schiff base derivatives, **6a-l**, were prepared by refluxing compounds **4a** and **4b** with proper (un) substituted isatin in ethanol for 14–18 h, giving **6a-l** in good yields. The validity of the structures of **6a-l** was verified by employing ^1^H NMR, ^13^C NMR, and elemental microanalysis experiments. The ^1^H NMR spectrum of compound **6k**, as a representative example, displayed five distinct singlet signals, which are characteristic of this compound. There is a singlet at δ 14.38 ppm which corresponds to two phenolic OH groups. There is another singlet at δ 11.52 ppm which corresponds to amidic NH groups. Additionally, there is a singlet at δ 10.93 ppm which corresponds to isatin NH groups. There is also a singlet at δ 3.95 ppm which corresponds to an aryl methylene group (Ar-CH_2_). Lastly, there is a singlet at δ 3.76 ppm which corresponds to six protons of two methoxy groups. Additionally, aromatic protons have distinct signals.

### 2.2 Biology

#### 2.2.1 *In vitro* antimicrobial activities

The antimicrobial effects of the currently synthesized Schiff bases **(6a-l)** were tested against two Gram-positive bacteria (*S. aureus* and *B. subtilis*), two Gram-negative bacteria (*E. coli* and *P. aeruginosa*), and two strains of fungus (*A. flavus* and *C. albicans*). The modified disk diffusion method (Alkhaldi et al., 2019; Manso et al., 2021) was used to determine the inhibition zones (IZ, mm/mL) and the minimal inhibitory concentration (MIC, nM). Ciprofloxacin and Fluconazole was used as positive controls. Tables 1–3 display the findings.

**TABLE 1:** Inhibition zone diameter (mm/mg) of compounds **6a-l** and reference drugs.

Sample	Inhibition zone (IZ) diameter (mm/mg)					
	Bacterial species				Fungi	
	(G^+^)		(G^−^)			
	*B. subtilis*	*S. aureus*	*E. Coli*	*P. aeruginosa*	*A. flavus*	*C. albicans*
**6a**	36	36	34	35	18	28
**6b**	26	27	26	26	11	18
**6c**	38	38	37	38	22	31
**6d**	30	31	30	30	13	20
**6e**	37	38	36	38	21	30
**6f**	34	35	35	32	16	25
**6g**	23	25	22	22	9.0	15
**6h**	45	48	45	45	31	36
**6i**	31	32	33	32	15	22
**6j**	40	41	40	40	25	33
**6k**	29	31	27	25	14	20
**6l**	43	46	43	42	29	35
**Ciprofloxacin**	40	40	40	40	NA	NA
**Fluconazole**	NA	NA	NA	NA	40	40

NA: no activity (8 mm), weak activity (8–15 mm), moderate activity (15–20 mm), strong activity (>20 mm), DMSO, as solvent (8 mm), and *Bacillus subtilis* (*B. subtilis*), *Staphylococcus aureus* (*S. aureus*), *Escherichia coli* (*E. coli*), *Pseudomonas aeruginosa* (*P. aeruginosa*), *Candida albicans* (*C. albicans*), and *Aspergillus flavus (A. flavus)*.

**TABLE 2:** MICs and MBCs of compounds **6c**, **6e**, **6h**, **6j**, and **6l**.

Compound	Bacterial species							
	(G^+^)				(G^−^)			
	*B. subtilis*		*S. aureus*		*E. coli*		*P. aeruginosa*	
	*MIC*	*MBC*	*MIC*	*MBC*	*MIC*	*MBC*	*MIC*	*MBC*
6c	33 ± 2	51 ± 3	29 ± 2	45 ± 2	66 ± 4	88 ± 6	65 ± 5	85 ± 6
6e	37 ± 2	58 ± 3	35 ± 2	54 ± 3	69 ± 4	95 ± 6	69 ± 5	97 ± 6
6 h	24 ± 2	38 ± 2	19 ± 1	28 ± 1	54 ± 3	74 ± 6	55 ± 4	74 ± 5
6j	31 ± 2	47 ± 2	25 ± 2	40 ± 2	62 ± 4	81 ± 6	61 ± 5	80 ± 6
6l	26 ± 2	43 ± 3	21 ± 2	33 ± 2	57 ± 4	77 ± 6	57 ± 4	78 ± 6
Ciprofloxacin	10 ± 1	19 ± 1	30 ± 2	45 ± 2	60 ± 4	90 ± 6	60 ± 5	90 ± 6

**TABLE 3:** MICs and MFCs of compounds **6c**, **6e**, **6h**, **6j**, and **6l**.

Compound	Fungi (µM)			
	*A. flavus*		*C. albicans*	
	*MIC*	*MFC*	*MIC*	*MFC*
6c	18.20 ± 1	35.90 ± 2	21.30 ± 1	44.80 ± 3
6e	19.50 ± 1	40.20 ± 3	22.90 ± 1	50.20 ± 3
6 h	14.50 ± 1	28.50 ± 1	18.20 ± 1	36.50 ± 2
6j	16.90 ± 1	33.90 ± 2	20.70 ± 1	41.50 ± 3
6l	15.60 ± 1	31.10 ± 2	19.50 ± 1	39.20 ± 3
Fluconazole	11.50 ± 1	22.80 ± 1	17.50 ± 1	35.00 ± 2

Based on the inhibition zones (IZ) measurements in Table 1, we can conclude that five Schiff bases (**6c**, **6e**, **6h**, **6j**, and **6l**) had inhibition zones that were either greater than or comparable to the inhibition zones of the reference medications (ciprofloxacin and fluconazole) against pathogenic organisms. Schiff bases **6a-l** were found to have strong antibacterial activity against both Gram-positive and Gram-negative bacteria, with inhibition zones (IZ) ranging from 22 to 48 mm for all pathogens tested, compared to Ciprofloxacin’s IZ of 40 mm. Moreover, Table 1 demonstrated that compounds **6h** and **6l** had superior efficacy compared to the reference ciprofloxacin against both gram-positive and gram-negative bacteria. In addition, most of the newly synthesized Schiff bases **6a-l** had IZ values between 11 and 36 mm against *A. flavus* and *C. albicans*, whereas fluconazole, a broad spectrum antifungal medication, had a value of 40 mm. We proceeded with the investigation to ascertain the minimal inhibitory concentrations (MIC, nM) of the potent Schiff bases (**6c**, **6e**, **6h**, **6j**, and **6l**). The results are displayed in Tables 2, 3.

#### 2.2.2 Minimum inhibitory concentration (MIC) assay

The most potent components **6c**, **6e**, **6h**, **6j**, and **6l** were evaluated for antibacterial activity using a twofold serial dilution method on a 96-well microtiter plate (Wiegand et al., 2008). Table 2 showed the MICs (nM) of these compounds against the tested bacteria, with ciprofloxacin as the reference medicine. The results of this *in vitro* assay test are consistent with the findings of the antimicrobial sensitivity test. Compound **6h** (R = Cl, X = H) was the most potent of the compounds examined, having MIC values of 19, 54, and 55 nM against *S. aureus*, *E. coli*, and *P. aeruginosa*. It displayed greater potency to ciprofloxacin against the investigated species but had a MIC value of 24 nM against *B. subtilis*, which is 2.5 times less effective than ciprofloxacin (MIC = 10 nM). Compound **6l** (R = NO_2_, X = H) exhibited the second highest activity. Its MIC values were comparable to those of ciprofloxacin against *E. coli* and *P. aeruginosa*, Table 2. However, it was 2.5 times less effective than ciprofloxacin against *B. subtilis*. Compounds **6h** and **6l** were 1.5 times more effective than ciprofloxacin against *S. aureus*, with MIC values of 19 and 21 nM, respectively, whereas ciprofloxacin had a MIC value of 30 nM. Compounds **6c** (R = Br, X = Cl) and **6j** (R = CH_3_, X = H) exhibited significant activity against the tested species, especially against *S. aureus*, with MICs values of 29 and 25 nM, respectively. These values were comparable to that of ciprofloxacin (MIC = 30 nM). Finally, compound **6e** (with R = OCH_3_ and X = Cl) demonstrated the lowest level of efficacy. It has lower antimicrobial activity than ciprofloxacin against all pathogens tested.

#### 2.2.3 Minimum bactericidal concentration (MBC) assay

The MBC differs from the MIC. The MIC test finds the lowest concentration of an antimicrobial agent that significantly inhibits growth, whereas the MBC identifies the lowest concentration that causes microbiological organisms to die. In contrast to the MBC, which does result in mortality, the MIC just inhibits, meaning that the antibacterial action does not cause death (Abdullahi et al., 2023). MBC is typically reported as MBC_50_, indicating that the antibiotic concentration kills 50% of the initial bacterial population (Sun et al., 2019).

In general, components **6c**, **6e**, **6h**, **6j**, and **6l** had strong bactericidal activity. The MBC values for Gram-positive bacteria ranged from 28 to 58 nM, whereas ciprofloxacin MBC values were 19 and 45 nM, Table 2. Compounds **6h** (R = Cl, X = H) and **6l** (R = NO_2_, X = H), the most potent antibacterial agents, displayed bactericidal activity of 28 and 33 nM, respectively, for *S. aureus*, compared to ciprofloxacin’s MBC value of 45 nM, making **6h** and **6l** more effective as bactericidal agents. Compound **6j** (R = CH_3_, X = H) scored third in bactericidal activity with MBC value of 40 nM against *S. aureus*, which was comparable to the reference ciprofloxacin. Regrettably, compounds **6h**, **6j**, and **6l** exhibited lower effectiveness as bactericidal agents compared to ciprofloxacin against *B. subtilis*. The MBC values for compounds **6h**, **6j**, and **6l** were 38, 47, and 43 nM, respectively, while ciprofloxacin had an MBC value of 19 nM.

In the case of Gram-negative bacteria, compounds **6h**, **6j**, and **6l** had higher MBC values than the other compounds examined. These compounds have MBC values of 74, 80, and 78 nM, making them more effective than ciprofloxacin (MBC = 90 nM) against both *E. Coli* and *P. aeruginosa* species. Again, compound **6e** (with R = OCH_3_ and X = Cl) was the least effective as bactericidal agents against all of the bacterial species tested.

#### 2.2.4 Antifungal assay

Compounds **6c**, **6e**, **6h**, **6j**, and **6l** were tested for antifungal activity with a twofold serial dilution method (Wiegand et al., 2008). Table 3 displays the MICs (µM) and MFCs (minimum fungicidal concentration, µM) of these derivatives against *A. flavus* and *C. albicans* fungus, using fluconazole as the reference medication. Overall, the investigated compounds displayed robust antifungal efficacy against the selected fungal species when compared to ciprofloxacin. Compounds **6c**, **6e**, **6h**, **6j**, and **6l** exhibited MIC values ranging from 14.50 to 19.50 µM against *A. flavus*, while fluconazole had a MIC value of 11.50 µM. Moreover, the tested compounds demonstrated MIC values ranging from 18.20 to 22.90 µM against *C. albicans*, while the reference fluconazole had a MIC of 17.50 µM.

Compound **6h** (R = Cl, X = H), the most potent antibacterial agent, was also the most potent antifungal agent, with MIC values of 14.50 µM against *A. flavus* and 18.20 µM against *C. albicans*, being comparable to the reference fluconazole, which had MIC values of 11.50 and 17.50 µM, respectively. Compound **6l (**R = NO_2_, X = H) demonstrated the second highest antifungal activity. It exhibited MIC values of 15.60 µM against *A. flavus* and 19.50 µM against *C. albicans*. Finally, Table 3 shows that all investigated compounds had fungicidal activity, with MFC/MIC ratios ranging around two.

#### 2.2.5 Antibacterial assay against multi-drug resistant strains

The most potent Schiff bases **6c**, **6e**, **6h**, **6j**, and **6l** were examined for their antibacterial efficacy against a panel of MDRB (multi-drug resistant bacteria), including one clinical strain *S. aureus* (ATCC 43300) (MRSA), two standard strain *E. coli* (ATCC BAA-196) and *P. aeruginosa* (ATCC BAA-2111). Norfloxacin, a broad-spectrum antibiotic, worked as positive control. Results are displayed in Table 4.

**TABLE 4:** MICs and MBCs of compounds **6c**, **6e**, **6h**, **6j**, and **6l** against MDRB strains.

Compound	*S. aureus ATCC 43300*		*E. coli ATCC-BAA-196*		*P. aeruginosa ATCC-BAA-2111*	
	*MIC*	*MBC*	*MIC*	*MBC*	*MIC*	*MBC*
**6c**	4.70 ± 0.20	7.10 ± 0.50	5.30 ± 0.20	8.20 ± 0.60	6.85 ± 0.50	9.20 ± 0.60
**6e**	5.90 ± 0.20	7.80 ± 0.50	6.25 ± 0.25	9.30 ± 0.60	7.15 ± 0.50	10.40 ± 0.70
**6h**	1.70 ± 0.10	3.20 ± 0.15	1.90 ± 0.10	3.70 ± 0.20	3.40 ± 0.20	5.80 ± 0.30
**6j**	3.20 ± 0.15	5.90 ± 0.25	3.90 ± 0.15	6.10 ± 0.25	5.60 ± 0.25	7.80 ± 0.30
**6l**	2.20 ± 0.10	4.50 ± 0.20	2.70 ± 0.10	4.80 ± 0.20	4.50 ± 0.20	6.50 ± 0.25
**Norfloxacin**	1.20 ± 0.10	2.80 ± 0.10	1.60 ± 0.10	3.50 ± 0.10	3.20 ± 0.10	4.70 ± 0.20

The MIC values for the tested compounds vary from 1.70 to 7.15 µM, while the MBC values range from 3.50 to 10.40 µM against MDRB bacteria. These values are compared to the MIC values of norfloxacin, which range from 1.20 to 3.20 µM, and the MBC values, which range from 2.80 to 4.70 µM. The majority of the investigated Schiff bases, compounds **6c**, **6e**, **6h**, **6j**, and **6l**, had substantial broad-spectrum effects on both Gram-positive and Gram-negative bacteria, either by inhibiting their growth or by killing them (bactericidal effect). Notably, compounds **6h** and **6l** show the most potent actions compared to other derivatives. Compound **6h** (R = Cl, X = H) showed a MIC value of 1.70 µM against the clinical strain *S. aureus* (ATCC 43300) (MRSA), making it an effective antibacterial agent. It was found to be approximately as potent as the reference drug norfloxacin, which had a MIC of 1.20 µM. Compound **6h**, on the other hand, exhibited an MBC of 3.90 µM, making it around 1.2 times more potent against the MRSA strain than the reference norfloxacin. Furthermore, compound **6h** demonstrated similar efficacy to norfloxacin against *E. coli* (ATCC BAA-196) and *P. aeruginosa* (ATCC BAA-2111), with MICs of 1.9 and 3.4 µM, respectively. Norfloxacin, on the other hand, showed MIC values of 1.6 and 3.2 µM. Additionally, compound **6l** (R = NO_2_, X = H) showed significant antibacterial activity against MDRB strains, with MIC values ranging from 2.20 to 4.50 µM and MBC values ranging from 4.50 to 6.50 µM. Based on the MBC/MIC ratio, we identified that all of the compounds tested had values less than two, indicating bactericidal activity.

#### 2.2.6 DNA gyrase and DHFR inhibitory assay

The inhibitory potency of derivatives **6h**, **6j**, and **6l**, the most potent antibacterial agents, against *E. coli* DNA gyrase and DHFR was determined using the *E. coli* DNA gyrase and DHFR assay (Durcik et al., 2018). The findings are shown as IC_50_ values for the investigated compounds and reference drugs (Table 5). The results of this assay complement those of the antibacterial activity investigation. Compounds **6h**, **6j**, and **6l** inhibited *E. coli* DNA gyrase at IC_50_ values of 79, 117, and 87 nM, respectively, compared to the reference novobiocin (IC_50_ = 170 nM). Compounds **6h**, **6j**, and **6l** exhibited greater potency compared to the reference compound novobiocin, with compounds **6h** and **6l** being twice as potent as novobiocin against DNA gyrase.

**TABLE 5:** IC_50_ values of **6h, 6j,** and **6l** against *E. coli* DNA gyrase and DHFR.

Compound	IC_50_ (nM)	IC_50_ (µM)
	*E. Coli* DNA gyrase	DHFR *E. Coli*
6 h	79 ± 5	3.80 ± 0.10
6j	117 ± 8	5.10 ± 0.20
6l	87 ± 6	4.25 ± 0.10
Novobiocin	170 ± 20	--
Trimethoprim	--	5.20 ± 0.20

Compounds **6h**, **6j**, and **6l** were further evaluated against the DHFR enzyme as indicated in Table 5. Compounds **6h** and **6l**, the most potent *E. coli* gyrase inhibitors, also demonstrated promising effects on DHFR. Compounds **6h** and **6l** have IC_50_ values of 3.80 µM and 4.25 µM, respectively. These values are much lower and more potent than trimethoprim’s IC_50_ value of 5.20 µM. Based on these observations, we may infer that both compounds **6h** and **6j** show promise as dual-target inhibitors against DNA gyrase and DHFR, especially after optimization.

#### 2.2.7 Antibiofilm assay

Bacterial biofilms provide health risks in hospitals, the food industry, and drinking water systems. In the current work, we investigated the antibiofilm activity of compound **6h**, the most potent derivative, against *S. aureus* using Microtiter plate assay for biofilm quantification (Antunes et al., 2010; Niu and Gilbert, 2004). The assay was performed with three different concentrations, the first of which was equivalent to the MIC of **6h** against *S. aureus* ATCC 43300 (Table 4), the second of which was equivalent to 1/2 MIC, and finally 1/4 MIC. The results were presented in Table 6 as Biofilm inhibition%.

**TABLE 6:** Antibiofilm assay of compound **6h**.

6 h	Biofilm inhibition %	SD (±)
1/4 of MIC	64	0.49
1/2 MIC	88	0.64
MIC	97	0.42

The results showed that **6h** has significant antibiofilm action, with biofilm inhibition percentage equal to 97 at the MIC dose. Compound **6h** inhibited biofilms by 88% and 64% at ½ and ¼ MIC levels, respectively.

Blank represented absorbance of media only.

Control represented absorbance of test organism without any treatment.

#### 2.2.8 Cell viability assay

This test examines the impact of compounds **6c**, **6e**, **6h**, **6j**, and **6l**, which are the most potent derivatives, on normal cell lines in order to assess the safety level of these compounds. The vitality of the investigated compounds was assessed using the MCF-10A cell line, which is a normal human mammary gland epithelial cell line. Following a 4-day incubation period on MCF-10A cells with a concentration of 50 µM for each compound being studied, the viability of the cells was assessed using the MTT test (Hisham et al., 2022; Mahmoud et al., 2022). The results from Table 7 indicate that none of the compounds tested exhibited cytotoxicity, and all compounds show a cell viability of more than 89% at a concentration of 50 µM.

**TABLE 7:** Cell viability assay of compounds **6c**, **6e**, **6h**, **6j**, and **6l**

Comp	Cell viability %
6c	91
6e	92
6 h	93
6j	92
6l	89

### 2.3 Docking studies

#### 2.3.1 Docking study of *E. Coli* DNA gyrase B

**FIGURE 4:**
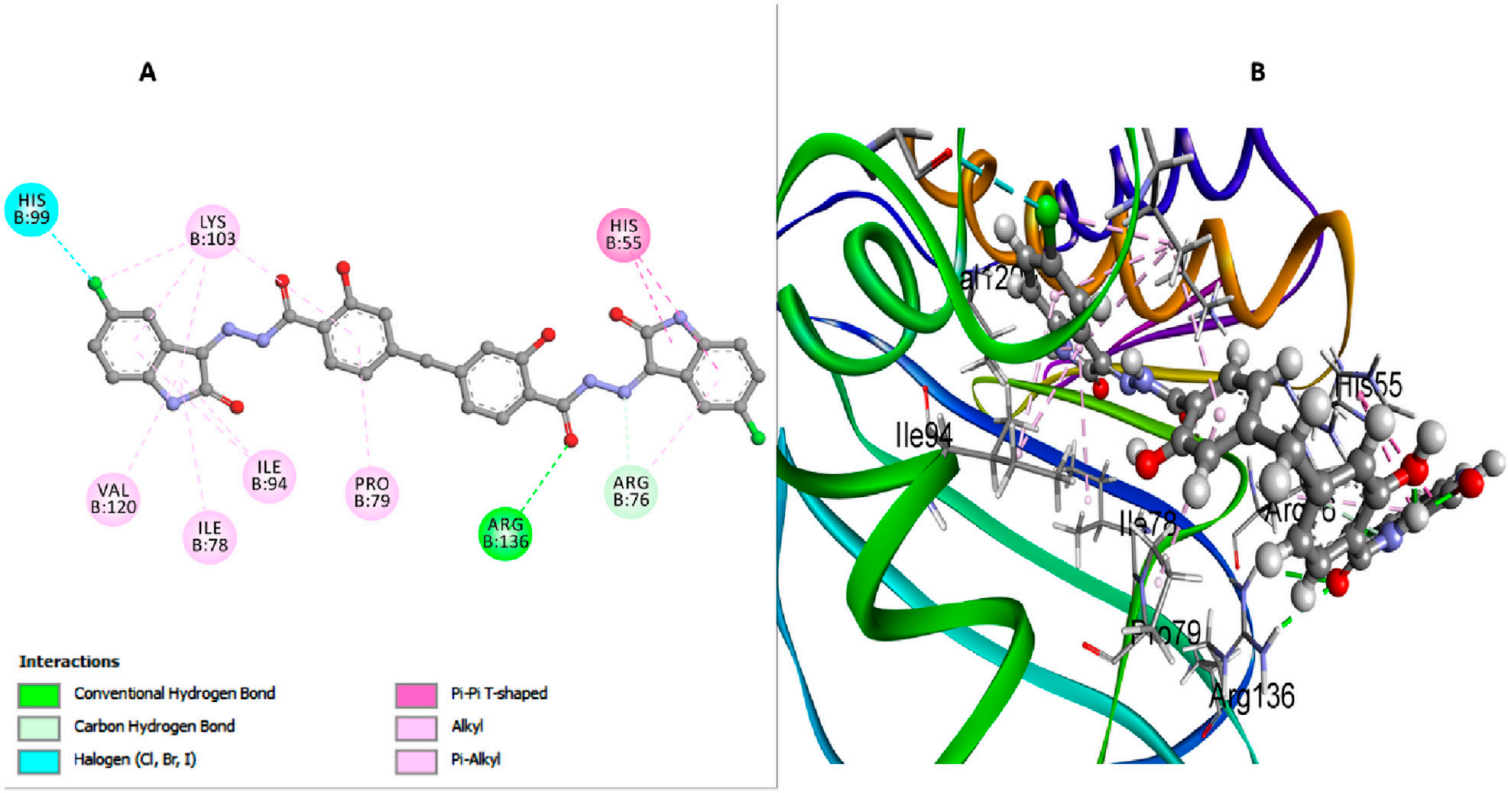
Docking representation models of compound **6h** within the binding site of *E. coli* DNA gyrase B protein; **(A)** 2D-docked model of compound **6h**; **(B)** 3D-docked model of compound **6h**.

This investigation performed an extensive computational docking analysis to determine the binding affinities of compounds **6b**, **6h**, and **6l** with *E. coli* DNA gyrase B and *E. coli* DHFR. Novobiocin served as the reference drug for *E. coli* DNA gyrase B, while trimethoprim was used as the reference for *E. coli* DHFR. Utilizing Discovery Studio software (Baroroh et al., 2023), the study provided an in-depth examination of the interaction mechanisms between these compounds and their respective target proteins. To ensure the accuracy and relevance of our investigation, we incorporated the crystallographic structure of the *E. coli* DNA gyrase B ligand complex (PDB ID: 4DUH) from the Protein Data Bank (Marinho et al., 2023). The OPLS-AA (Optimized Potentials for Liquid Simulations - All Atom) force field was utilized during the energy minimization process for the molecular systems under examination. Implementing this force field was crucial for achieving conformational stability of the molecular structures, thereby enhancing the precision and reliability of our computational analyses (Hazarika et al.; Kumar H. et al., 2023). Prior to initiating the docking procedure, the protein structure underwent comprehensive preparation to ensure its accuracy. This preparation included protonation, a critical step that significantly contributed to the robustness and reliability of the subsequent docking analysis. A comparative analysis between docking scores and *in vitro* activity levels of *E. coli* DNA gyrase B for the compounds studied revealed a direct correlation. Compound **6h**, which demonstrated the highest *in vitro* activity against *E. coli* DNA gyrase B, achieved a docking score of −7.88 kcal/mol. In contrast, compound **6b**, with lower *in vitro* antibacterial activity and a smaller inhibition zone (IZ) measurement compared to the ciprofloxacin, had a docking score of −5.78 kcal/mol. Compound **6l** recorded a docking score of −7.51 kcal/mol, reflecting its relatively strong binding affinity and correlating with its moderate *in vitro* activity. Moreover, Novobiocin, used as a reference drug in the study, displayed a docking score of −7.26 kcal/mol, indicating its considerable binding affinity. In analyzing interactions between the tested compounds and the *E. coli* DNA gyrase B protein, compound **6h** has exhibited notable binding characteristics. An important hydrogen bond interaction between the carbonyl oxygen of the salicylate moiety and Arg136 stabilizes **6h** within the active site. This hydrogen bond is crucial as it anchors the compound, preventing its dissociation from the active site and enhancing its inhibitory efficacy (Figure 4). Moreover, the absence of a bulky atom, such as the 3-chloro group on the di-salicylate nucleus of **6h**, facilitates the free rotation of the two salicylate moieties around the methylene axis. This rotational freedom is crucial as it enables the compound to adopt a conformation that effectively blocks the entrance to the active site. By fitting more snugly into the binding pocket, **6h** can comprehensively obstruct substrate access, thereby enhancing its ability to inhibit *E. coli* gyrase activity. The isatin nucleus within **6h** significantly enhances its binding affinity. The docking interactions reveal that the isatin moiety forms essential interactions with several amino acid residues within the active site. Notably, the π-π T-shaped interactions with residues such as His55 and Lys103 underscore the crucial role of the isatin nucleus in stabilizing the enzyme-inhibitor complex. The aromatic nature of isatin allows for these non-covalent interactions, which strengthen the overall binding and stability of **6h** within the active site (Figure 4). Furthermore, the presence of substituents like the 5-chloro group on the isatin nucleus of **6h** further amplifies its binding affinity. The 5-chloro substituent increases hydrophobic interactions with the active site residues and contributes to the electronic distribution of the molecule. The electron-withdrawing nature of the 5-chloro group enhances interactions with residues like His99. Additionally, this substituent helps position the isatin moiety in an optimal orientation for interaction with the enzyme, thereby boosting the inhibitory potential of **6h** (Figure 4).

**FIGURE 5:**
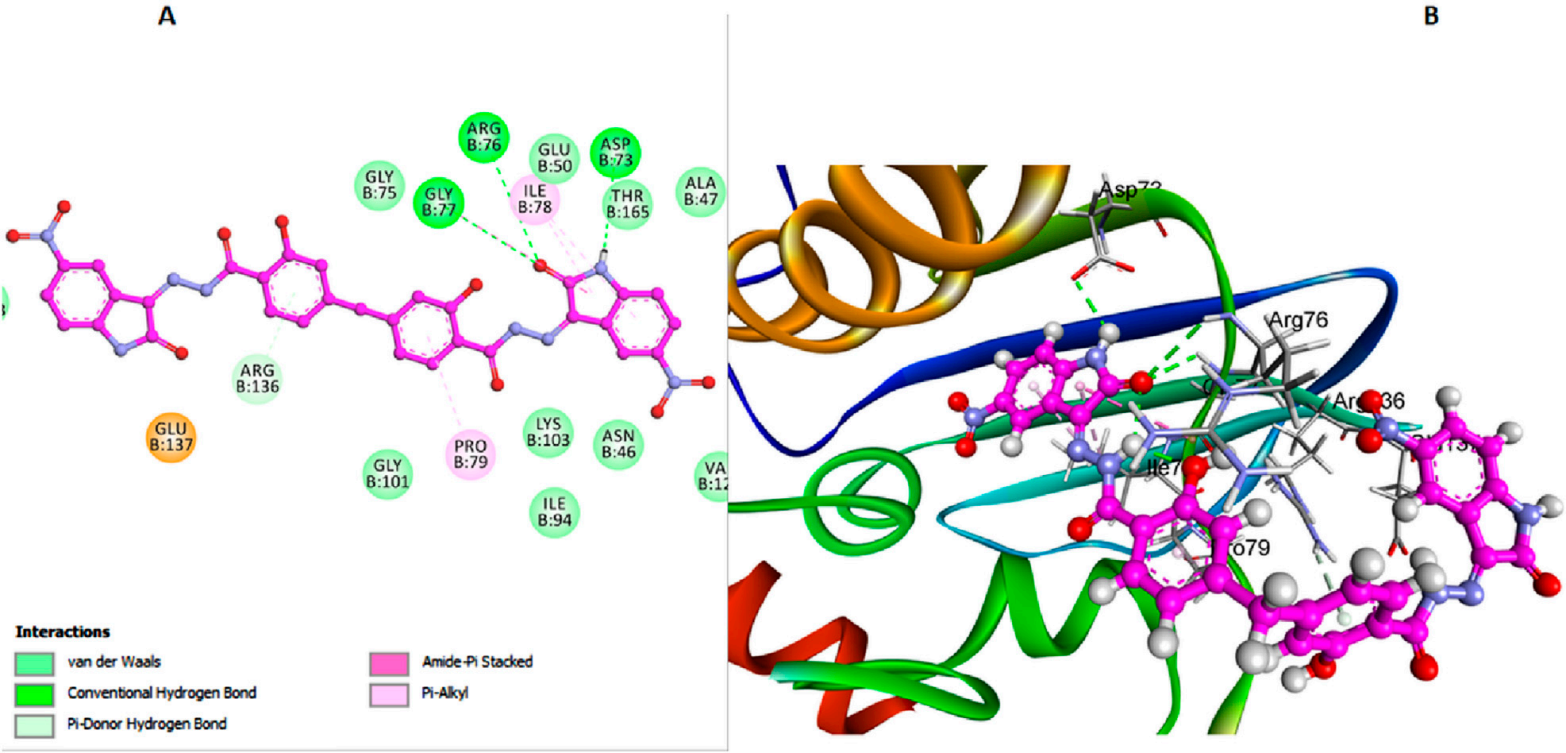
Docking representation models of compound **6l** within the binding site of *E. coli* DNA gyrase B protein; **(A)** 2D-docked model of compound **6l**; **(B)** 3D-docked model of compound **6l**.

In contrast, compound **6l**, which also lacks the bulky 3-chloro group on the di-salicylate nucleus like 6 h, exhibits less binding affinity due to fewer interactions with the active site. The absence of the 3-chloro group allows **6l** to have a certain degree of rotational freedom, but not as effectively as **6h**. This results in a less snug fit within the binding pocket, making it less efficient in blocking substrate access (Figure 5). Arg136 forms a Pi-donor hydrogen bond with the salicylate moiety, while Gly77 and Arg76 interact with the isatin moiety. Thr165 and Asp73 contribute to stabilizing the binding conformation through additional hydrogen bonds.

**FIGURE 6:**
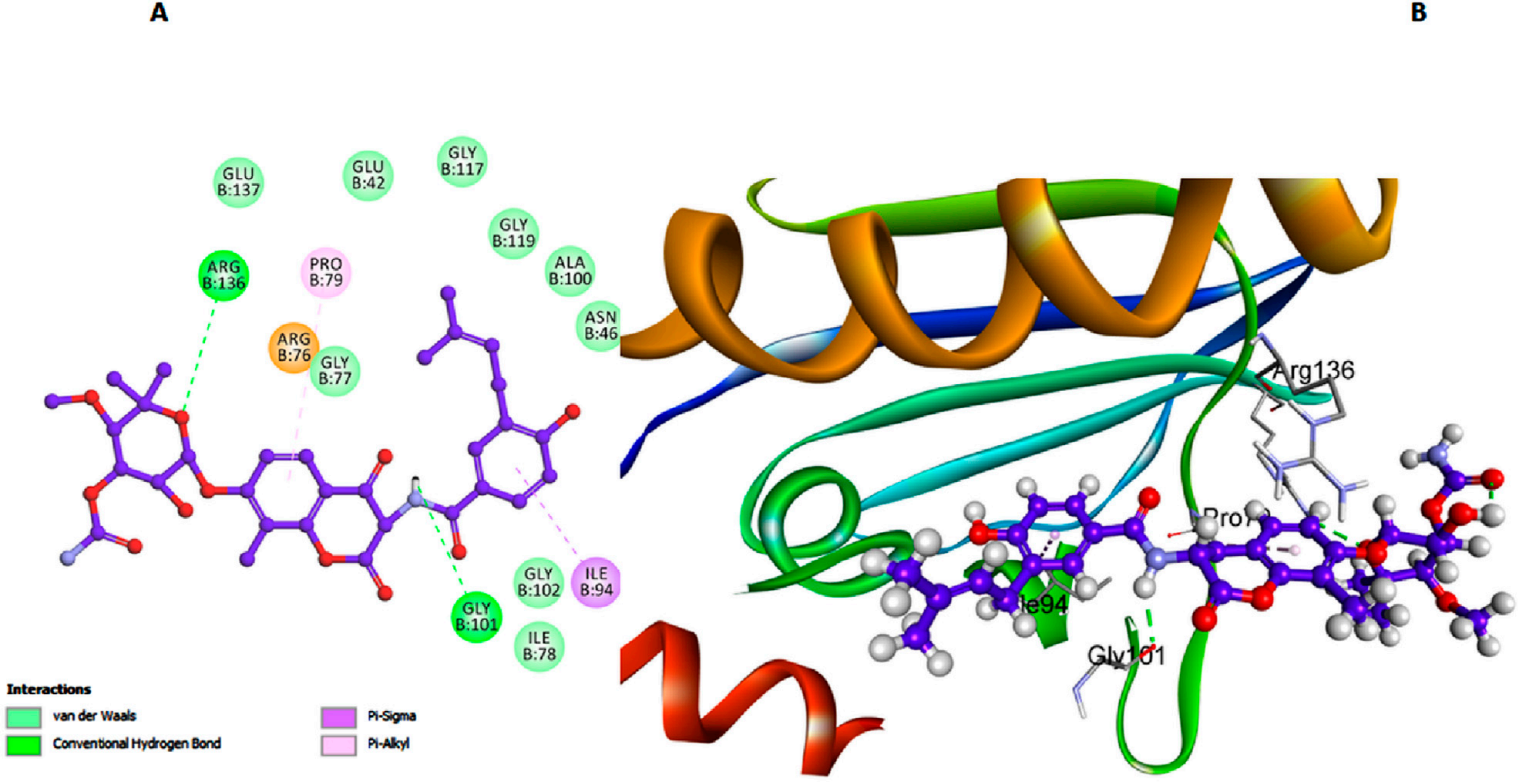
Docking representation models of Novobiocin within the binding site of *E. coli* DNA gyrase B protein; **(A)** 2D-docked model of Novobiocin; **(B)** 3D-docked model of Novobiocin.

In addition, Novobiocin exhibited notable interactions with the *E. coli* DNA gyrase B protein, with its amide nitrogen functioning as a hydrogen bond acceptor in conjunction with Gly 101, as depicted in Figure 6. However, this significant interaction results in Novobiocin having a comparatively lower binding affinity to the active site than compounds **6h** and **6l**. This reduced affinity can be attributed to the lack of additional stabilizing interactions that compounds **6h** and **6l** possess, such as the π-π interactions with His55 and Lys103 in compound **6h** and the hydrophobic interactions facilitated by the 5-chloro group in compound **6h** (Figure 4). Moreover, the rigid structure of Novobiocin, which might limit its flexibility and ability to adopt an optimal conformation within the active site. This structural rigidity can result in fewer contacts with key residues within the binding pocket, leading to weaker overall binding interactions (Figure 6).

**FIGURE 7:**
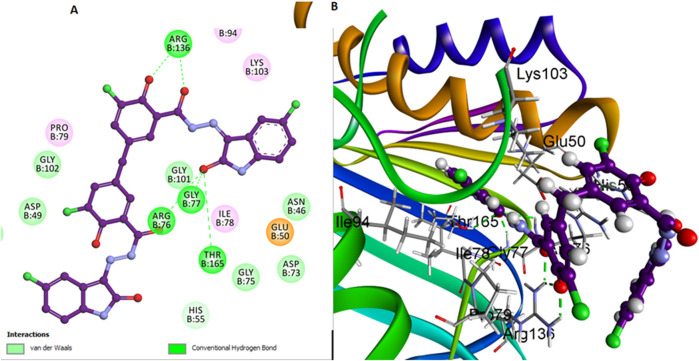
Docking representation models of compound **6b** within the binding site of *E. coli* DNA gyrase B protein; **(A)** 2D-docked model of compound **6b**; **(B)** 3D-docked model of compound **6b**.

On the other hand, compound **6b** features a bulky atom, the 3-chloro group on the di-salicylate nucleus, which significantly influences its interaction with *E. coli* gyrase. The presence of the 3-chloro group restricts the free rotation of the salicylate moieties around the methylene axis. This restriction limits the conformational flexibility of **6b**, potentially reducing its ability to snugly fit into the binding pocket and block substrate access as effectively as **6h** (Figure 7).

Comparative analysis between compounds **6h**, **6l**, and **6b** reveals insights into their flexibility and stability. Compound **6h** benefits from the absence of bulky substituents, allowing for greater conformational flexibility and a snugger fit within the active site, leading to potentially higher inhibitory efficacy. Compound **6l**, while also lacking the bulky 3-chloro group on salicylate, forms fewer interactions within the active site, resulting in reduced binding affinity and inhibitory potency compared to **6h**. On the other hand, compound **6b**, with its bulky 3-chloro group on salicylate, faces a steric hindrance that reduces its conformational adaptability.

#### 2.3.2 Docking study of *E. Coli* DHFR

**FIGURE 8:**
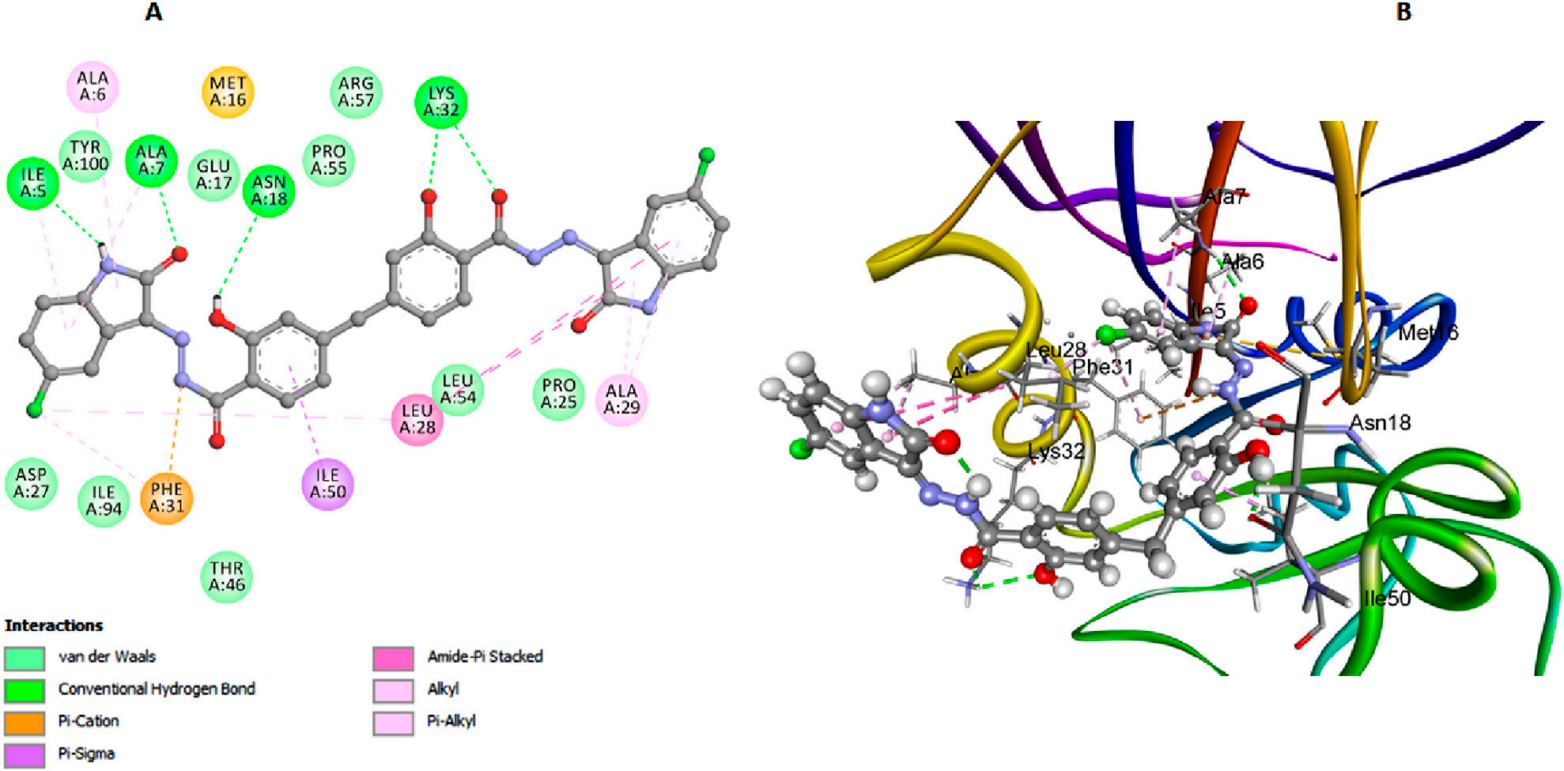
Docking representation models of compound **6h** within the binding site of *E. coli* DHFR enzyme; **(A)** 2D-docked model of compound **6h**; **(B)** 3D-docked model of compound **6h**.

In our study on *E. coli* DHFR, we utilized the crystallographic structure of its ligand complex (PDB ID: 6CXK) from the Protein Data Bank as a foundational framework for computational modeling (Bhati, 2024). Compound **6h**, which demonstrated the highest *in vitro* activity against *E. coli* DHFR, achieved a docking score of −7.42 kcal/mol. Similarly, compound **6l**, with nearly comparable *in vitro* activity, recorded a docking score of −7.28 kcal/mol. Trimethoprim, used as the reference compound in this study, exhibited a docking score of −6.94 kcal/mol. The significance of the docking score of trimethoprim lies in its role as a benchmark, highlighting that compounds **6h** and **6l** exhibit stronger binding affinities, as evidenced by their lower docking scores, which correlates with their higher *in vitro* activities against *E. coli* DHFR. In the comprehensive analysis focusing on the interaction profiles between the investigated compounds and *E. coli* DHFR, compound **6h** exhibited significant binding characteristics. The docking interactions of compound **6h** with the DHFR *E. coli* enzyme provide detailed insights into its inhibitory potential. The carbonyl and phenolic oxygens of the salicylate moiety form two crucial hydrogen bonds with Lys32, anchoring **6h** within the active site (Figure 8). Additionally, π-stacked interactions with Leu28 and Leu54 further enhance the binding affinity of **6h**, while hydrogen bonds with Ile5 and Asn18 help stabilize its overall conformation within the active site. These interactions highlight the importance of specific residues in stabilizing the binding of compound **6h**, allowing it to fit snugly and effectively block substrate access, thereby enhancing its inhibitory efficacy against the DHFR *E. coli* enzyme.

**FIGURE 9:**
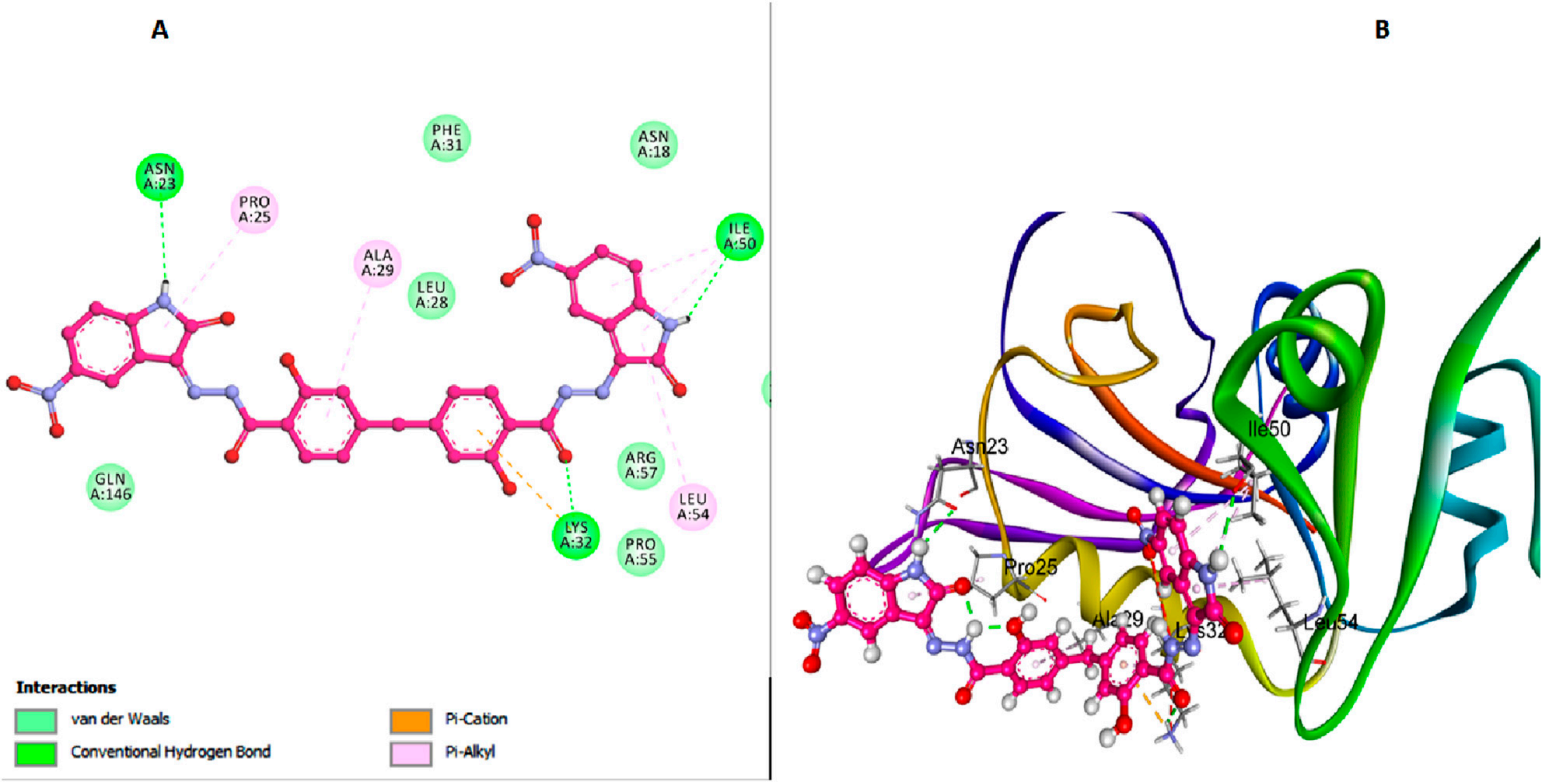
Docking representation models of compound **6l** within the binding site of *E. coli* DHFR enzyme; **(A)** 2D-docked model of compound **6l**; **(B)** 3D-docked model of compound **6L**.

Similarly, compound **6l** exhibits a binding affinity to DHFR *E. coli* enzyme that is comparable to **6h**. The docking interactions of **6l** reveal several key interactions contributing to its stability and inhibitory potential. The carbonyl oxygen of the salicylate moiety in **6l** forms a crucial hydrogen bond with Lys32, mirroring the interaction seen with **6h** (Figure 9). Pi-cation interactions between the aromatic ring of the salicylate moiety and Ala29 further stabilize the enzyme-inhibitor complex. Additionally, isatin-pi stacked interactions with Leu54, and Pro25 enhance the binding affinity of **6l**, while hydrogen bonds with Asn23 and Ile50 contribute to the overall stability of the compound within the active site. These interactions allow **6l** to occupy the DHFR active site effectively, forming stable bonds with key residues and blocking substrate access, thus inhibiting enzyme activity.

**FIGURE 10:**
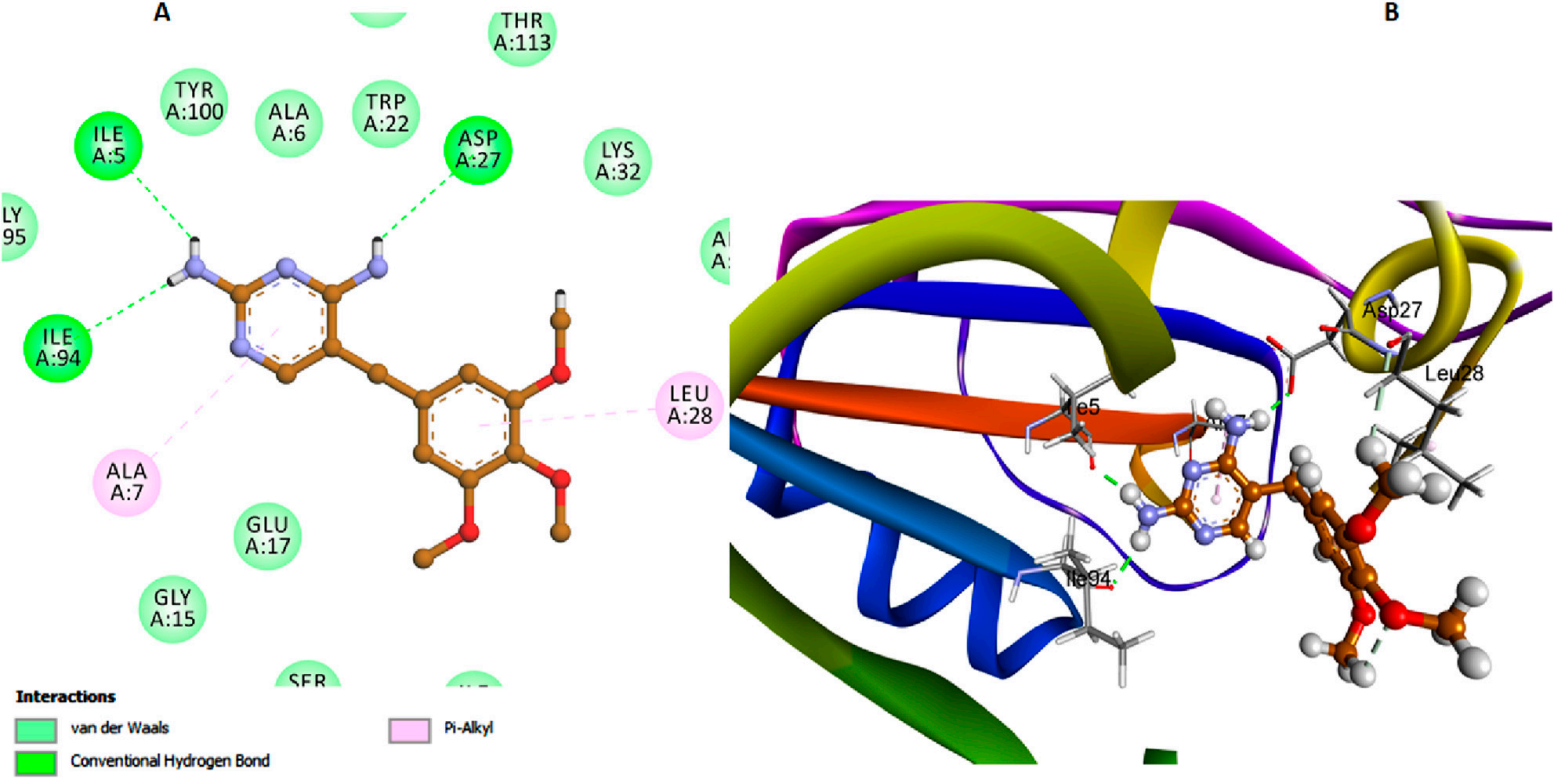
Docking representation models of Trimethoprim within the binding site of *E. coli* DHFR enzyme; **(A)** 2D-docked model of Trimethoprim; **(B)** 3D-docked model of Trimethoprim.

Trimethoprim exhibited notable interactions with the *E. coli* DHFR enzyme, with its amino nitrogen functioning as a hydrogen bond acceptor in conjunction with Asp27, as depicted in Figure 10. However, this significant interaction results in Trimethoprim having a comparatively lower binding affinity to the active site than compounds **6h** and **6l**. This reduced affinity can be attributed to the lack of additional stabilizing interactions that compounds **6h** and **6l** possess, such as the π-π stacking interactions with Ala29 and Lys32 of di-salicylate and also, the network interactions facilitated by the isatin nucleus.

This dual modeling against *E. coli* DNA gyrase B and DHFR enzymes has crucial implications for antibacterial drug development. Particularly, the favorable docking poses of **6h** suggest its potential as dual inhibitor against *E. coli* DNA gyrase B and DHFR enzymes.

In conclusion, The molecular docking results provided crucial insights into the binding affinities and interaction mechanisms of the compounds with *E.* coli DNA gyrase B and DHFR enzymes. As summarized in Table 8, compound **6h** demonstrated the strongest binding affinity due to key hydrogen bonds and π-π interactions with active site residues, making it particularly effective in blocking substrate access. Comparatively, compound **6l** and reference drugs like Novobiocin and Trimethoprim exhibited fewer stabilizing interactions, resulting in relatively lower binding affinities.

**TABLE 8:** Binding affinity and molecular interactions of compounds **6b**, **6h** and **6l** with *E*. coli DNA gyrase B and DHFR enzymes in molecular docking studies.

Compound	Target enzyme	Docking score (kcal/mol)	Hydrogen bonding	Amino acids involved	Other important parameters
**6h**	*E. coli* DNA gyrase B	−7.88	Carbonyl oxygen of salicylate ↔ Arg136	His55, Lys103, Arg136	π-π interactions with His55 and Lys103, hydrophobic interactions by 5-chloro group, flexibility due to no 3-chloro group
	*E. coli* DHFR	−7.42	Carbonyl and phenolic oxygens ↔ Lys32	Leu28, Leu54, Ile5, Asn18	π-stacked with Leu28 and Leu54, stabilization through hydrogen bonds with Ile5 and Asn18
**6l**	*E. coli* DNA gyrase B	−7.51	Salicylate ↔ Arg136	Gly77, Arg76, Thr165, Asp73	Lacks 3-chloro, π-donor hydrogen bond with Arg136, reduced binding due to fewer active site interactions
	*E. coli* DHFR	−7.28	Salicylate oxygen ↔ Lys32	Ala29, Leu54, Pro25, Asn23, Ile50	π-cation interaction with Ala29, isatin π-stacked with Leu54, Pro25
**6b**	*E. coli* DNA gyrase B	−5.78	-	-	Presence of bulky 3-chloro group on di-salicylate nucleus reduces flexibility and binding effectiveness
**Novobiocin**	*E. coli* DNA gyrase B	−7.26	Amide nitrogen ↔ Gly101	-	Limited interactions due to rigidity, lacks flexibility for optimal binding
**Trimethoprim**	*E. coli* DHFR	−6.94	Amino nitrogen ↔ Asp27	Ala29, Lys32	Lower affinity due to fewer stabilizing interactions compared to 6h and 6l

### 2.4 Molecular dynamics (MD) simulation against *E. coli* DNA gyrase B

**FIGURE 11:**
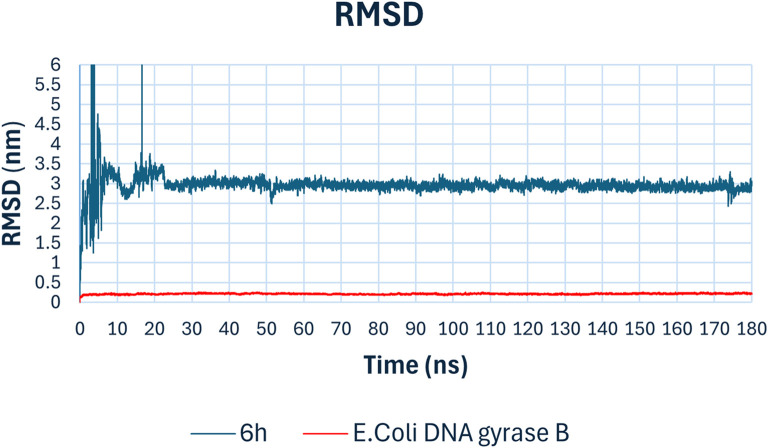
Rmsd analysis of compound **6h** with *E. coli* DNA gyrase B.

To validate the docking study results, a molecular dynamics (MD) simulation was performed using GROMACS 2023 (Vieira et al., 2023). The MD simulations for compound **6h**, the top hit against *E. coli* DNA gyrase B enzyme, provided comprehensive insights into the stability and binding interactions of the compound within the active site over 180 ns simulation period. The key parameters analyzed include RMSD, RMSF, radius of gyration, hydrogen bonds, and potential energy. The Root Mean Square Deviation (RMSD) plot (Figure 11) indicates the stability of the compound-enzyme complex over the 180 ns simulation period. The RMSD of the backbone atoms of *E. coli* DNA gyrase B (red line) remained stable around 0.5 nm, signifying minimal fluctuations and indicating that the protein structure was well-maintained throughout the simulation. The RMSD of compound **6h** (blue line) showed initial fluctuations within the first 20 ns, stabilizing thereafter around 2.5 nm. This initial fluctuation is typical as the compound adjusts within the binding pocket, after which the stable RMSD suggests a consistent binding conformation.

**FIGURE 12:**
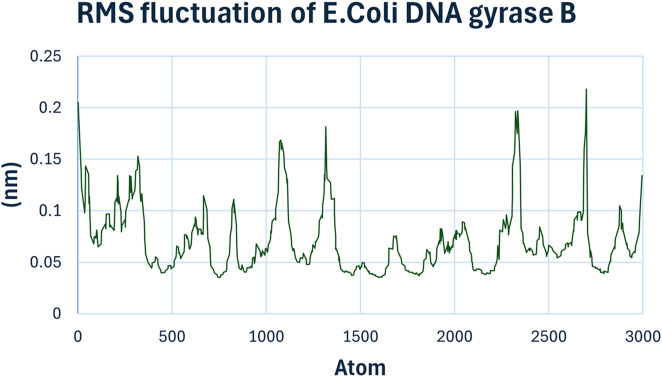
RMSF Analysis of compound **6h** with *E. coli* DNA Gyrase B.

The Root Mean Square Fluctuation (RMSF) chart (Figure 12) provides insights into the flexibility of individual amino acid residues. Peaks in the RMSF plot indicate regions of higher flexibility. Most residues showed low fluctuations, suggesting a rigid binding environment. However, certain residues around the binding site exhibited slightly higher fluctuations, indicating their involvement in dynamic interactions with compound **6h**.

**FIGURE 13:**
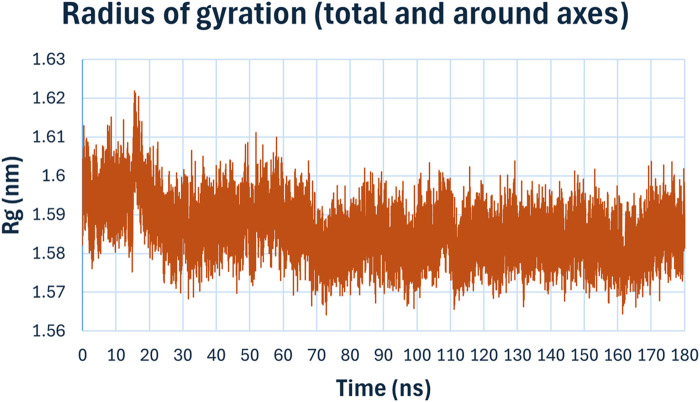
Radius of gyration (Rg) of compound **6h** with *E. coli* DNA Gyrase B.

The radius of gyration (Rg), (Figure 13), measures the compactness of the protein structure over time. The Rg values for the complex remained consistently around 1.59 nm, with minor fluctuations, indicating that the overall compactness and tertiary structure of the protein were maintained during the simulation. This suggests that the binding compound **6h** does not induce significant conformational changes in the protein structure.

**FIGURE 14:**
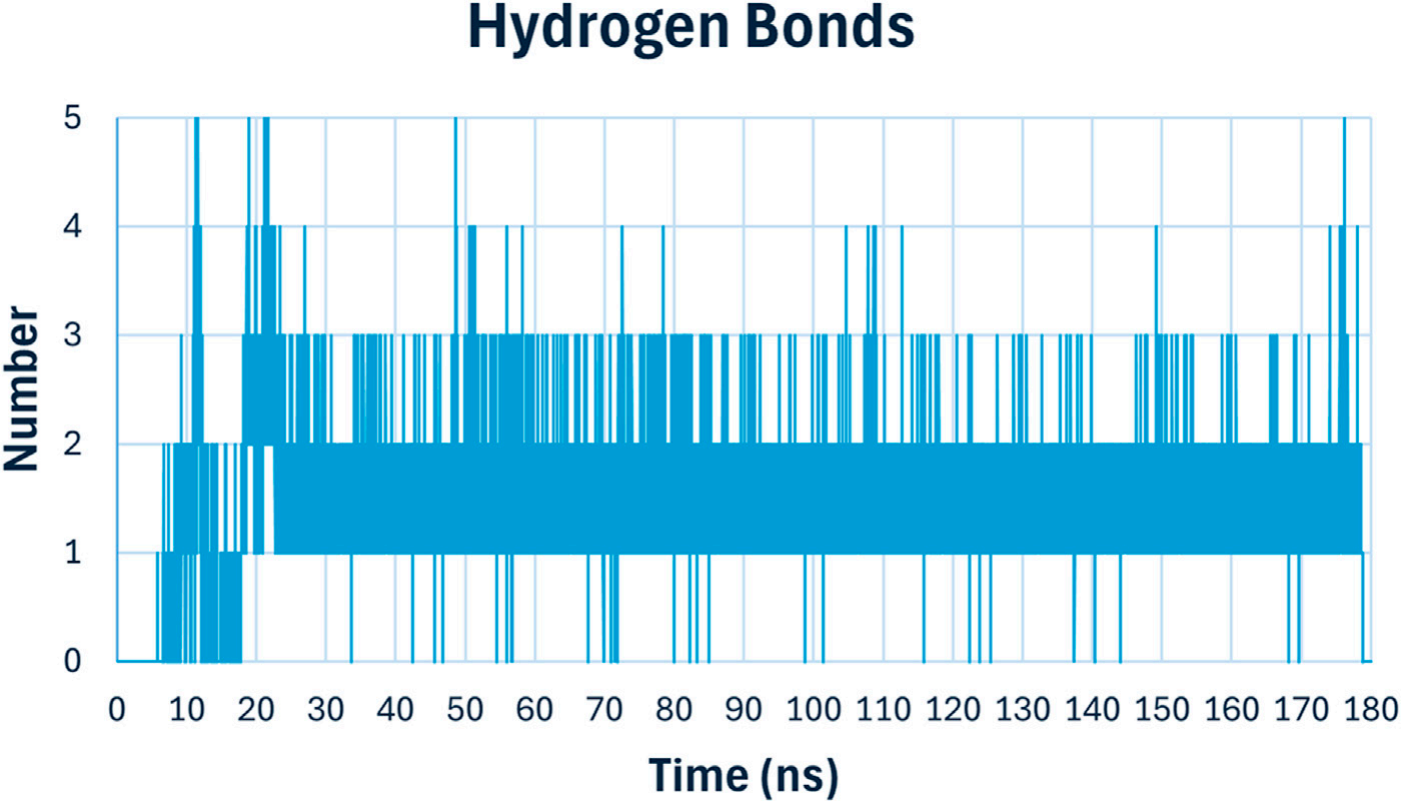
Hydrogen bonding analysis of compound **6h** with *E. coli* DNA Gyrase B.

Hydrogen bonding analysis (Figure 14) is crucial for understanding the stability and specificity of ligand binding. The number of hydrogen bonds between compound **6h** and *E. coli* DNA gyrase B fluctuated between 1 and 4 throughout the simulation, stabilizing mostly around 2 hydrogen bonds. These persistent hydrogen bonds are indicative of strong and stable interactions between the ligand and the protein, contributing to the high binding affinity observed in docking studies.

**FIGURE 15:**
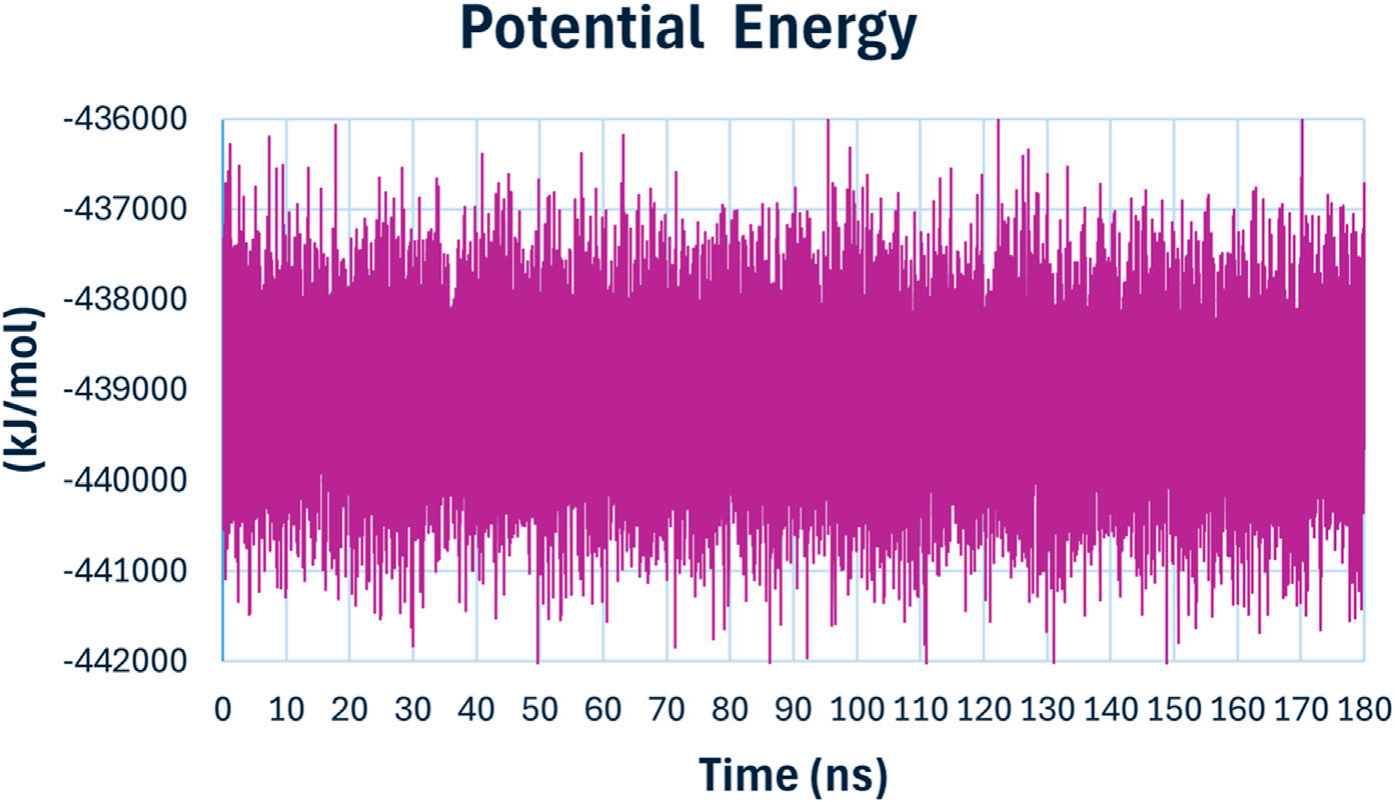
The potential energy of compound **6h** with *E. coli* DNA Gyrase B.

The potential energy (Figure 15) of the system remained relatively stable throughout the simulation, fluctuating around −438000 kJ/mol. The stability in potential energy further confirms the stability of the protein-ligand complex during the MD simulation, indicating that no significant energetic disturbances occurred, and the system was equilibrated.

The MD simulation results demonstrate that compound **6h** forms a stable complex with *E. coli* DNA gyrase B. The consistent RMSD, stable radius of gyration, persistent hydrogen bonding, and stable potential energy all point towards a robust binding interaction. These findings support the potential of compound **6h** as a promising lead compound for further development as an antibacterial agent targeting replication mechanisms. The observed molecular interactions and stability underscore its efficacy and provide a strong foundation for subsequent experimental validation and optimization studies.

## 3 Conclusion

Twelve compounds **(6a-l)** were developed by combining disalicylic acid methylene hydrazide with various isatin derivatives. The antibacterial activity of newly synthesized compounds **6a-l** was assessed against a variety of gram-negative and gram-positive bacterial strains, as well as fungal species. These novel targets were tested for DNA gyrase and DHFR inhibitory activities. The results showed that Compounds **6h** and **6l** were the most potent antibacterial agents, with MIC and MBC values comparable to or even lower than the reference Ciprofloxacin. Compound **6h** had a promising MIC value against the clinical strain *S. aureus* (ATCC 43300) (MRSA), indicating that it is an efficient antibacterial agent. It was shown to be about as potent as the reference medication, Norfloxacin. DNA gyrase and DHFR inhibitory experiments revealed that compounds **6h** and **6l** were twice as potent as novobiocin against DNA gyrase and more potent as DHFR inhibitors than the standard trimethoprim. Based on these data, we may conclude that both compounds **6h** and **6j** show promise as dual-target inhibitors of DNA gyrase and DHFR, especially following optimization. Additionally, compound 6 h demonstrated substantial antibiofilm effect, with a biofilm inhibition percentage of 97 at the MIC level. Docking experiments demonstrated that compound **6h** has a high affinity for both the DNA gyrase and the DHFR enzyme. MD simulations lasting 180 nanoseconds demonstrated compound **6h**′s stability and binding interactions in the active region of DNA gyrase. The computational results, together with the promising antibacterial activity exhibited in laboratory tests for compound **6h**, indicate that it has the potential to be developed as a lead compound in combating bacterial infections that are resistant to many treatments. Subsequent study will focus on improving the molecular structure of **6h** to optimize its pharmacokinetic characteristics and efficacy in biological systems.

## Data Availability

The original contributions presented in the study are included in the article/Supplementary Material, further inquiries can be directed to the corresponding authors.
